# Bis(2-chloro-1,10-phenanthroline-κ^2^ *N*,*N*′)(thio­cyanato-κ*N*)zinc (2-chloro-1,10-phenanthroline-κ^2^ *N*,*N*′)tris­(thio­cyanato-κ*N*)zincate

**DOI:** 10.1107/S1600536812002280

**Published:** 2012-01-25

**Authors:** Qing Hua Liu, Shu Lian Liu, Yan Hui Chi, Jing Min Shi

**Affiliations:** a College of Chemistry, Chemical Engineering and Materials Science, Shandong Normal University, Jinan 250014, People’s Republic of China; b School for Cadres of Shandong Bureau of Quality and Technical Supervision, Jinan 250014, People’s Republic of China

## Abstract

The asymmetric unit of the title compound, [Zn(NCS)(C_12_H_7_ClN_2_)_2_][Zn(NCS)_3_(C_12_H_7_ClN_2_)], contains two cations and two anions. In the cations, the Zn^II^ ions have distorted trigonal–bipyramidal environments formed by four N atoms from two 2-chloro-1,10-phenanthroline (cphen) ligands and one N atom from a thio­cyanate ligand. The Zn^II^ atoms in the complex anions also have distorted trigonal–bipyramidal environments, formed by two N atoms from a cphen ligand and three N atoms from three thio­cyanato ligands. The crystal packing exhibits π–π inter­actions between the rings of the cphen ligands [shortest centroid–centroid distance = 3.586 (5) Å] and short inter­molecular S⋯Cl [3.395 (5) Å] and S⋯S [3.440 (4) Å] contacts.

## Related literature

For a related structure, see: Li *et al.* (2008 ▶).
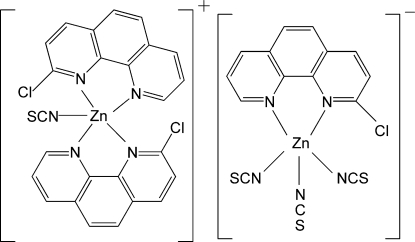


## Experimental

### 

#### Crystal data

[Zn(NCS)(C_12_H_7_ClN_2_)_2_][Zn(NCS)_3_(C_12_H_7_ClN_2_)]
*M* *_r_* = 1007.00
Triclinic, 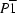

*a* = 15.259 (2) Å
*b* = 17.957 (3) Å
*c* = 18.665 (3) Å
α = 111.163 (2)°
β = 98.201 (2)°
γ = 111.273 (2)°
*V* = 4218.5 (10) Å^3^
*Z* = 4
Mo *K*α radiation
μ = 1.57 mm^−1^
*T* = 298 K
0.50 × 0.26 × 0.19 mm

#### Data collection

Bruker SMART APEX CCD diffractometer
Absorption correction: multi-scan (*SADABS*; Sheldrick, 1996 ▶) *T*_min_ = 0.508, *T*_max_ = 0.755
24046 measured reflections
17019 independent reflections
9850 reflections with *I* > 2σ(*I*)
*R*_int_ = 0.022

#### Refinement

*R*[*F* ^2^ > 2σ(*F* ^2^)] = 0.072
*wR*(*F* ^2^) = 0.250
*S* = 1.09
17019 reflections
1063 parameters
H-atom parameters constrained
Δρ_max_ = 1.87 e Å^−3^
Δρ_min_ = −0.86 e Å^−3^

### 

Data collection: *SMART* (Bruker, 2007 ▶); cell refinement: *SAINT* (Bruker, 2007 ▶); data reduction: *SAINT*; program(s) used to solve structure: *SHELXTL* (Sheldrick, 2008 ▶); program(s) used to refine structure: *SHELXTL*; molecular graphics: *SHELXTL*; software used to prepare material for publication: *SHELXTL*.

## Supplementary Material

Crystal structure: contains datablock(s) I, global. DOI: 10.1107/S1600536812002280/cv5234sup1.cif

Structure factors: contains datablock(s) I. DOI: 10.1107/S1600536812002280/cv5234Isup2.hkl

Additional supplementary materials:  crystallographic information; 3D view; checkCIF report

## Figures and Tables

**Fig. 1.**
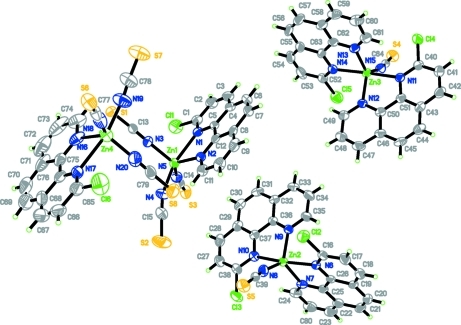
The content of asymmetric unit of (I) showing the atomic numbering and 30% probability displacement ellipsoids.

## supplementary crystallographic information

### Comment

Derivatives of 1,10-phenanthroline play an important role in modern coordination chemistry and many complexes have been reported with these compounds as ligands [for example a closely related Cd complex, see Li et al. (2008)]. To our knowledge, there are no publications dealing with complexes with 2-chloro-1,10-phenanthroline (cphen) ligands . Herein we report the first crystal structure of the title compound (I) with cphen ligands.

The asymmetric unit of (I) (Fig. 1) contains two cationic and two anionic complexes, respectively. The cationic complex involves two cphen neutral ligands and one thiocyanate anionic ligand, whereas the anionic complex involves one cphen neutral ligands and three thiocyanate anionic ligands. In both complexes, the zinc(II) ions assume distorted trigonal bipyramidal coordinated geometry.

In the crystal, there is a slipped π-π stacking interaction between the adjacent two complexes, the relevant distances being Cg1···Cg2^i^ 3.586 (5) Å and Cg1···Cg2^i^_perp_ = 3.468 Å [symmetry code: (i) x, -1+y, z], where Cg1 and Cg2 are centroids of C19-C22/C25/C26 and C55-C58/C62/C63, respectively and Cg1···Cg2^i^_perp_ is the perpendicular distance from Cg1 ring to Cg2^i^ ring. The crystal packing exhibits also short contacts between the adjacent complexes, which include S8···Cl6^i^ with the separation of 3.395 (5) Å and S4···S4^ii^ with the separation of 3.44o(4) Å [symmetry codes: (i) 1-x, 1-y, 2-z; (ii) 2-x, 2-y, -z].

### Experimental

A 5 mL H_2_O solution of NaNCS (0.0232 g, 0.286 mmol) was added into 15 mL methanol solution containing Zn(ClO_4_).6H_2_O (0.0678 g, 0.182 mmol) and 2-chloro-1,10-phenanthroline (0.0436 g, 0.203 mmol), and the mixed solution was stirred for a few minutes. The yellow single crystals were obtained after the filtrate had been allowed to stand at room temperature for about one week.

### Refinement

All H atoms were placed in calculated positions, and refined as riding, with C—H = 0.93 Å, *U*_iso_ = 1.2*U*_eq_(C).

### Crystal data

**Table:**

[Zn(NCS)(C_12_H_7_ClN_2_)_2_][Zn(NCS)_3_(C_12_H_7_ClN_2_)]	*Z* = 4
*M**_r_* = 1007.00	*F*(000) = 2024
Triclinic, *P*1	*D*_x_ = 1.586 Mg m^−^^3^
Hall symbol: -P 1	Mo *K*α radiation, λ = 0.71073 Å
*a* = 15.259 (2) Å	Cell parameters from 4824 reflections
*b* = 17.957 (3) Å	θ = 2.2–23.6°
*c* = 18.665 (3) Å	µ = 1.57 mm^−^^1^
α = 111.163 (2)°	*T* = 298 K
β = 98.201 (2)°	Prism, yellow
γ = 111.273 (2)°	0.50 × 0.26 × 0.19 mm
*V* = 4218.5 (10) Å^3^	

### Data collection

**Table:**

Bruker SMART APEX CCD diffractometer	17019 independent reflections
Radiation source: fine-focus sealed tube	9850 reflections with *I* > 2σ(*I*)
graphite	*R*_int_ = 0.022
φ and ω scans	θ_max_ = 26.5°, θ_min_ = 1.2°
Absorption correction: multi-scan (*SADABS*; Sheldrick, 1996)	*h* = −11→19
*T*_min_ = 0.507, *T*_max_ = 0.755	*k* = −22→21
24046 measured reflections	*l* = −22→23

### Refinement

**Table:**

Refinement on *F*^2^	Primary atom site location: structure-invariant direct methods
Least-squares matrix: full	Secondary atom site location: difference Fourier map
*R*[*F*^2^ > 2σ(*F*^2^)] = 0.072	Hydrogen site location: inferred from neighbouring sites
*wR*(*F*^2^) = 0.250	H-atom parameters constrained
*S* = 1.09	*w* = 1/[σ^2^(*F*_o_^2^) + (0.1377*P*)^2^] where *P* = (*F*_o_^2^ + 2*F*_c_^2^)/3
17019 reflections	(Δ/σ)_max_ = 0.003
1063 parameters	Δρ_max_ = 1.87 e Å^−^^3^
0 restraints	Δρ_min_ = −0.86 e Å^−^^3^

### Special details

**Table:**

Geometry. All esds (except the esd in the dihedral angle between two l.s. planes) are estimated using the full covariance matrix. The cell esds are taken into account individually in the estimation of esds in distances, angles and torsion angles; correlations between esds in cell parameters are only used when they are defined by crystal symmetry. An approximate (isotropic) treatment of cell esds is used for estimating esds involving l.s. planes.
Refinement. Refinement of F^2^ against ALL reflections. The weighted R-factor wR and goodness of fit S are based on F^2^, conventional R-factors R are based on F, with F set to zero for negative F^2^. The threshold expression of F^2^ > 2sigma(F^2^) is used only for calculating R-factors(gt) etc. and is not relevant to the choice of reflections for refinement. R-factors based on F^2^ are statistically about twice as large as those based on F, and R- factors based on ALL data will be even larger.

### Fractional atomic coordinates and isotropic or equivalent isotropic displacement parameters (Å^2^)

**Table:**

	*x*	*y*	*z*	*U*_iso_*/*U*_eq_	
C1	0.2824 (5)	0.5342 (4)	0.3899 (4)	0.0648 (16)	
C2	0.3350 (6)	0.6056 (5)	0.3752 (5)	0.080 (2)	
H2	0.3084	0.6127	0.3315	0.096*	
C3	0.4264 (7)	0.6639 (5)	0.4276 (5)	0.085 (2)	
H3	0.4638	0.7129	0.4203	0.102*	
C4	0.4665 (5)	0.6526 (4)	0.4925 (5)	0.0681 (18)	
C5	0.4065 (4)	0.5748 (4)	0.4987 (3)	0.0484 (13)	
C6	0.5612 (5)	0.7150 (5)	0.5539 (5)	0.082 (2)	
H6	0.5992	0.7675	0.5518	0.098*	
C7	0.5945 (5)	0.6993 (4)	0.6122 (5)	0.076 (2)	
H7	0.6564	0.7405	0.6501	0.091*	
C8	0.5384 (4)	0.6198 (4)	0.6201 (4)	0.0600 (16)	
C9	0.5728 (5)	0.5984 (5)	0.6802 (4)	0.0702 (19)	
H9	0.6345	0.6373	0.7191	0.084*	
C10	0.5164 (5)	0.5219 (5)	0.6813 (4)	0.074 (2)	
H10	0.5395	0.5078	0.7211	0.088*	
C11	0.4235 (5)	0.4630 (5)	0.6231 (4)	0.0618 (16)	
H11	0.3860	0.4098	0.6246	0.074*	
C12	0.4436 (4)	0.5571 (4)	0.5622 (3)	0.0487 (13)	
C13	0.1184 (4)	0.4884 (4)	0.5682 (4)	0.0522 (14)	
C14	0.1733 (4)	0.2743 (4)	0.3041 (4)	0.0568 (15)	
C15	0.2224 (5)	0.2467 (5)	0.5365 (4)	0.0755 (19)	
C16	0.8025 (4)	0.2594 (4)	0.4061 (4)	0.0575 (15)	
C17	0.9029 (5)	0.2887 (4)	0.4401 (4)	0.0730 (19)	
H17	0.9386	0.3374	0.4907	0.088*	
C18	0.9469 (5)	0.2437 (5)	0.3969 (5)	0.076 (2)	
H18	1.0141	0.2617	0.4182	0.091*	
C19	0.8929 (5)	0.1706 (4)	0.3209 (4)	0.0618 (17)	
C20	0.9347 (5)	0.1229 (5)	0.2743 (6)	0.086 (2)	
H20	1.0013	0.1379	0.2939	0.103*	
C21	0.8782 (6)	0.0540 (5)	0.1995 (6)	0.084 (2)	
H21	0.9077	0.0236	0.1682	0.101*	
C22	0.7749 (6)	0.0269 (4)	0.1679 (4)	0.0697 (19)	
C23	0.7161 (7)	−0.0416 (5)	0.0938 (5)	0.090 (2)	
H23	0.7435	−0.0732	0.0612	0.108*	
C24	0.5771 (6)	−0.0158 (4)	0.1178 (4)	0.0721 (19)	
H24	0.5098	−0.0318	0.1007	0.087*	
C25	0.7306 (5)	0.0739 (4)	0.2146 (4)	0.0588 (16)	
C26	0.7909 (4)	0.1473 (4)	0.2911 (4)	0.0530 (14)	
C27	0.2556 (5)	−0.0070 (5)	0.1240 (4)	0.079 (2)	
H27	0.1998	−0.0601	0.1094	0.095*	
C28	0.2470 (5)	0.0556 (5)	0.1058 (4)	0.076 (2)	
H28	0.1848	0.0464	0.0794	0.091*	
C29	0.3325 (4)	0.1372 (4)	0.1265 (3)	0.0613 (16)	
C30	0.3320 (5)	0.2051 (5)	0.1067 (4)	0.0682 (18)	
H30	0.2722	0.1990	0.0789	0.082*	
C31	0.4166 (5)	0.2795 (4)	0.1270 (4)	0.0674 (18)	
H31	0.4144	0.3226	0.1114	0.081*	
C32	0.5083 (4)	0.2922 (4)	0.1720 (3)	0.0535 (14)	
C33	0.5985 (5)	0.3676 (4)	0.1938 (4)	0.0670 (17)	
H33	0.6007	0.4114	0.1777	0.080*	
C34	0.6809 (5)	0.3751 (4)	0.2379 (4)	0.075 (2)	
H34	0.7405	0.4254	0.2537	0.091*	
C35	0.6793 (5)	0.3094 (4)	0.2605 (4)	0.0632 (16)	
H35	0.7378	0.3170	0.2920	0.076*	
C36	0.5122 (4)	0.2262 (4)	0.1934 (3)	0.0478 (13)	
C37	0.4229 (4)	0.1454 (4)	0.1685 (3)	0.0497 (13)	
C38	0.3475 (5)	0.0069 (4)	0.1645 (4)	0.0637 (16)	
C39	0.5369 (4)	0.1117 (3)	0.4123 (4)	0.0481 (13)	
C40	1.1683 (5)	0.9785 (4)	0.3232 (4)	0.0623 (16)	
C41	1.2631 (5)	0.9911 (5)	0.3599 (4)	0.077 (2)	
H41	1.3194	1.0426	0.3716	0.092*	
C42	1.2704 (5)	0.9261 (5)	0.3778 (4)	0.077 (2)	
H42	1.3323	0.9327	0.4014	0.092*	
C43	1.1837 (5)	0.8484 (4)	0.3604 (4)	0.0603 (15)	
C44	1.1850 (5)	0.7793 (5)	0.3789 (4)	0.0716 (19)	
H44	1.2453	0.7836	0.4033	0.086*	
C45	1.1023 (6)	0.7085 (5)	0.3626 (4)	0.0682 (18)	
H45	1.1052	0.6635	0.3748	0.082*	
C46	1.0087 (5)	0.7008 (4)	0.3262 (4)	0.0576 (15)	
C47	0.9139 (6)	0.6257 (5)	0.3077 (4)	0.080 (2)	
H47	0.9121	0.5788	0.3189	0.096*	
C48	0.8298 (6)	0.6251 (5)	0.2745 (5)	0.088 (2)	
H48	0.7691	0.5784	0.2639	0.105*	
C49	0.8333 (5)	0.6947 (4)	0.2556 (4)	0.0737 (19)	
H49	0.7739	0.6923	0.2317	0.088*	
C50	1.0038 (4)	0.7674 (4)	0.3060 (3)	0.0528 (14)	
C51	1.0948 (4)	0.8444 (4)	0.3251 (3)	0.0507 (13)	
C52	0.6988 (5)	0.7632 (4)	0.1335 (5)	0.0709 (18)	
C53	0.5988 (5)	0.7485 (5)	0.1151 (6)	0.093 (3)	
H53	0.5513	0.7028	0.0665	0.112*	
C54	0.5751 (6)	0.8050 (6)	0.1724 (6)	0.093 (3)	
H54	0.5103	0.7976	0.1621	0.112*	
C55	0.6448 (5)	0.8715 (5)	0.2441 (5)	0.077 (2)	
C56	0.6226 (6)	0.9306 (6)	0.3052 (6)	0.090 (3)	
H56	0.5580	0.9238	0.2970	0.107*	
C57	0.6935 (7)	0.9957 (6)	0.3744 (6)	0.101 (3)	
H57	0.6771	1.0329	0.4136	0.121*	
C58	0.7933 (6)	1.0091 (5)	0.3888 (5)	0.076 (2)	
C59	0.8723 (7)	1.0760 (5)	0.4590 (5)	0.084 (2)	
H59	0.8601	1.1156	0.4998	0.101*	
C60	0.9656 (7)	1.0846 (5)	0.4692 (4)	0.086 (2)	
H60	1.0176	1.1290	0.5158	0.103*	
C61	0.9802 (5)	1.0238 (4)	0.4067 (4)	0.0692 (18)	
H61	1.0439	1.0286	0.4135	0.083*	
C62	0.8173 (5)	0.9505 (4)	0.3288 (4)	0.0589 (16)	
C63	0.7406 (4)	0.8801 (4)	0.2556 (4)	0.0593 (16)	
C64	0.9585 (4)	0.8887 (3)	0.0948 (3)	0.0470 (13)	
C65	0.2177 (6)	0.4476 (6)	1.0854 (5)	0.092 (2)	
C66	0.1588 (9)	0.3701 (7)	1.0898 (7)	0.115 (4)	
H66	0.1847	0.3556	1.1281	0.138*	
C67	0.0709 (10)	0.3190 (8)	1.0428 (8)	0.130 (4)	
H67	0.0323	0.2680	1.0470	0.156*	
C68	0.0327 (7)	0.3405 (5)	0.9843 (7)	0.106 (3)	
C69	−0.0626 (10)	0.2874 (9)	0.9254 (9)	0.145 (6)	
H69	−0.1016	0.2314	0.9210	0.174*	
C70	−0.0950 (7)	0.3134 (7)	0.8802 (8)	0.135 (6)	
H70	−0.1600	0.2778	0.8459	0.162*	
C71	−0.0377 (6)	0.3971 (7)	0.8767 (5)	0.108 (3)	
C72	−0.0736 (9)	0.4256 (9)	0.8261 (7)	0.152 (6)	
H72	−0.1382	0.3917	0.7908	0.183*	
C73	−0.0141 (8)	0.5035 (9)	0.8282 (5)	0.134 (5)	
H73	−0.0390	0.5224	0.7933	0.161*	
C74	0.0879 (7)	0.5600 (6)	0.8827 (4)	0.105 (3)	
H74	0.1281	0.6138	0.8835	0.126*	
C75	0.0602 (5)	0.4484 (5)	0.9317 (4)	0.077 (2)	
C76	0.0954 (5)	0.4217 (5)	0.9845 (4)	0.0688 (18)	
C77	0.3394 (4)	0.7169 (5)	1.1937 (4)	0.0706 (19)	
C78	0.3222 (7)	0.7666 (5)	0.9678 (5)	0.103 (3)	
C79	0.3816 (5)	0.5064 (6)	0.9168 (6)	0.086 (2)	
C80	0.6174 (7)	−0.0649 (5)	0.0661 (4)	0.085 (2)	
H80	0.5777	−0.1115	0.0151	0.103*	
Cl1	0.16301 (16)	0.46523 (16)	0.32717 (13)	0.1020 (7)	
Cl2	0.74689 (14)	0.31683 (12)	0.45881 (11)	0.0812 (5)	
Cl3	0.35886 (14)	−0.07416 (12)	0.18773 (13)	0.0900 (6)	
Cl4	1.15993 (13)	1.06089 (12)	0.30016 (13)	0.0844 (6)	
Cl5	0.73356 (15)	0.69534 (13)	0.06346 (12)	0.0952 (6)	
Cl6	0.33635 (19)	0.5119 (2)	1.14981 (18)	0.1489 (12)	
N1	0.3153 (3)	0.5167 (3)	0.4477 (3)	0.0505 (11)	
N2	0.3872 (3)	0.4811 (3)	0.5656 (3)	0.0465 (10)	
N3	0.1606 (4)	0.4540 (3)	0.5345 (3)	0.0616 (13)	
N4	0.2169 (5)	0.3023 (4)	0.5230 (4)	0.0810 (17)	
N5	0.1934 (4)	0.3158 (3)	0.3719 (3)	0.0639 (14)	
N6	0.7469 (3)	0.1914 (3)	0.3335 (3)	0.0492 (11)	
N7	0.6330 (4)	0.0516 (3)	0.1891 (3)	0.0538 (12)	
N8	0.5426 (4)	0.1148 (4)	0.3526 (3)	0.0676 (14)	
N9	0.5959 (3)	0.2353 (3)	0.2384 (3)	0.0484 (11)	
N10	0.4312 (3)	0.0814 (3)	0.1876 (3)	0.0559 (12)	
N11	1.0863 (3)	0.9088 (3)	0.3071 (3)	0.0515 (11)	
N12	0.9171 (4)	0.7637 (3)	0.2702 (3)	0.0548 (12)	
N13	0.9100 (4)	0.9591 (3)	0.3378 (3)	0.0557 (12)	
N14	0.7671 (4)	0.8252 (3)	0.2002 (3)	0.0577 (12)	
N15	0.9545 (4)	0.8768 (4)	0.1506 (3)	0.0643 (13)	
N16	0.1203 (5)	0.5284 (4)	0.9327 (3)	0.0778 (17)	
N17	0.1874 (4)	0.4730 (4)	1.0351 (3)	0.0678 (14)	
N18	0.3140 (5)	0.6769 (4)	1.1246 (4)	0.094 (2)	
N19	0.3062 (6)	0.7036 (5)	0.9772 (4)	0.116 (3)	
N20	0.3457 (5)	0.5429 (5)	0.9565 (4)	0.092 (2)	
S1	0.05930 (13)	0.53437 (12)	0.61849 (11)	0.0681 (5)	
S2	0.2339 (2)	0.16631 (18)	0.55444 (19)	0.1389 (11)	
S3	0.14738 (14)	0.21493 (14)	0.20727 (11)	0.0834 (6)	
S4	0.96500 (15)	0.90772 (14)	0.01809 (11)	0.0812 (6)	
S5	0.52714 (14)	0.10423 (14)	0.49362 (11)	0.0793 (5)	
S6	0.37306 (16)	0.77112 (14)	1.28828 (11)	0.0882 (6)	
S7	0.3501 (2)	0.85263 (16)	0.95253 (15)	0.1311 (11)	
S8	0.4327 (2)	0.4559 (2)	0.8587 (2)	0.1379 (12)	
Zn1	0.24234 (5)	0.40029 (4)	0.48672 (4)	0.0514 (2)	
Zn2	0.58525 (5)	0.13220 (4)	0.26516 (4)	0.0498 (2)	
Zn3	0.92815 (5)	0.86803 (4)	0.24576 (4)	0.0503 (2)	
Zn4	0.26864 (6)	0.59848 (5)	1.00670 (5)	0.0736 (3)	

### Atomic displacement parameters (Å^2^)

**Table:**

	*U*^11^	*U*^22^	*U*^33^	*U*^12^	*U*^13^	*U*^23^
C1	0.068 (4)	0.070 (4)	0.065 (4)	0.040 (3)	0.017 (3)	0.030 (3)
C2	0.099 (6)	0.093 (6)	0.089 (5)	0.057 (5)	0.042 (5)	0.063 (5)
C3	0.109 (6)	0.076 (5)	0.125 (7)	0.060 (5)	0.070 (6)	0.070 (5)
C4	0.073 (4)	0.053 (4)	0.101 (5)	0.038 (3)	0.046 (4)	0.040 (4)
C5	0.049 (3)	0.042 (3)	0.057 (3)	0.026 (3)	0.021 (3)	0.018 (3)
C6	0.066 (5)	0.058 (4)	0.132 (7)	0.027 (4)	0.043 (5)	0.050 (5)
C7	0.045 (4)	0.049 (4)	0.102 (6)	0.011 (3)	0.017 (4)	0.014 (4)
C8	0.044 (3)	0.063 (4)	0.063 (4)	0.031 (3)	0.020 (3)	0.011 (3)
C9	0.049 (4)	0.082 (5)	0.050 (4)	0.028 (4)	0.001 (3)	0.006 (3)
C10	0.071 (4)	0.105 (6)	0.053 (4)	0.049 (4)	0.013 (3)	0.037 (4)
C11	0.058 (4)	0.078 (4)	0.057 (4)	0.034 (3)	0.015 (3)	0.037 (3)
C12	0.042 (3)	0.048 (3)	0.051 (3)	0.027 (3)	0.017 (2)	0.009 (3)
C13	0.044 (3)	0.054 (3)	0.061 (4)	0.019 (3)	0.014 (3)	0.033 (3)
C14	0.042 (3)	0.057 (4)	0.065 (4)	0.014 (3)	0.015 (3)	0.030 (3)
C15	0.075 (5)	0.073 (5)	0.068 (4)	0.021 (4)	0.020 (3)	0.034 (4)
C16	0.052 (3)	0.045 (3)	0.070 (4)	0.016 (3)	0.016 (3)	0.028 (3)
C17	0.058 (4)	0.063 (4)	0.080 (5)	0.013 (3)	0.005 (3)	0.034 (4)
C18	0.053 (4)	0.071 (5)	0.111 (6)	0.024 (4)	0.021 (4)	0.053 (5)
C19	0.069 (4)	0.057 (4)	0.097 (5)	0.040 (3)	0.045 (4)	0.054 (4)
C20	0.072 (5)	0.086 (5)	0.148 (8)	0.049 (4)	0.064 (5)	0.077 (6)
C21	0.100 (6)	0.087 (5)	0.131 (7)	0.068 (5)	0.083 (6)	0.071 (5)
C22	0.094 (5)	0.061 (4)	0.085 (5)	0.046 (4)	0.057 (4)	0.042 (4)
C23	0.132 (7)	0.084 (6)	0.096 (6)	0.068 (6)	0.070 (6)	0.050 (5)
C24	0.100 (5)	0.057 (4)	0.061 (4)	0.035 (4)	0.029 (4)	0.027 (3)
C25	0.077 (4)	0.052 (4)	0.081 (4)	0.039 (3)	0.049 (4)	0.045 (3)
C26	0.061 (4)	0.045 (3)	0.079 (4)	0.031 (3)	0.040 (3)	0.040 (3)
C27	0.054 (4)	0.067 (5)	0.087 (5)	0.010 (3)	0.011 (4)	0.027 (4)
C28	0.047 (4)	0.084 (5)	0.068 (4)	0.021 (4)	0.005 (3)	0.018 (4)
C29	0.064 (4)	0.069 (4)	0.045 (3)	0.034 (3)	0.016 (3)	0.015 (3)
C30	0.075 (5)	0.089 (5)	0.046 (3)	0.052 (4)	0.013 (3)	0.021 (3)
C31	0.095 (5)	0.066 (4)	0.056 (4)	0.053 (4)	0.019 (4)	0.028 (3)
C32	0.067 (4)	0.053 (3)	0.050 (3)	0.035 (3)	0.019 (3)	0.024 (3)
C33	0.083 (5)	0.048 (4)	0.074 (4)	0.031 (3)	0.021 (4)	0.032 (3)
C34	0.071 (5)	0.054 (4)	0.094 (5)	0.015 (3)	0.023 (4)	0.040 (4)
C35	0.060 (4)	0.054 (4)	0.073 (4)	0.022 (3)	0.012 (3)	0.033 (3)
C36	0.055 (3)	0.046 (3)	0.040 (3)	0.022 (3)	0.017 (2)	0.016 (2)
C37	0.058 (3)	0.054 (3)	0.038 (3)	0.028 (3)	0.017 (2)	0.018 (3)
C38	0.059 (4)	0.054 (4)	0.063 (4)	0.013 (3)	0.016 (3)	0.024 (3)
C39	0.037 (3)	0.042 (3)	0.059 (4)	0.016 (2)	0.009 (2)	0.021 (3)
C40	0.065 (4)	0.060 (4)	0.073 (4)	0.033 (3)	0.028 (3)	0.033 (3)
C41	0.058 (4)	0.069 (5)	0.094 (5)	0.027 (3)	0.016 (4)	0.031 (4)
C42	0.068 (4)	0.083 (5)	0.080 (5)	0.046 (4)	0.007 (4)	0.030 (4)
C43	0.064 (4)	0.072 (4)	0.054 (3)	0.041 (3)	0.018 (3)	0.027 (3)
C44	0.084 (5)	0.085 (5)	0.057 (4)	0.055 (4)	0.012 (3)	0.031 (4)
C45	0.098 (5)	0.069 (4)	0.066 (4)	0.059 (4)	0.027 (4)	0.036 (4)
C46	0.080 (4)	0.055 (4)	0.057 (4)	0.040 (3)	0.032 (3)	0.031 (3)
C47	0.112 (6)	0.069 (5)	0.092 (5)	0.051 (4)	0.043 (5)	0.057 (4)
C48	0.084 (5)	0.077 (5)	0.132 (7)	0.039 (4)	0.041 (5)	0.072 (5)
C49	0.073 (4)	0.070 (4)	0.103 (5)	0.039 (4)	0.036 (4)	0.055 (4)
C50	0.071 (4)	0.056 (4)	0.047 (3)	0.037 (3)	0.026 (3)	0.028 (3)
C51	0.062 (4)	0.058 (3)	0.048 (3)	0.035 (3)	0.027 (3)	0.028 (3)
C52	0.067 (4)	0.060 (4)	0.097 (5)	0.027 (4)	0.030 (4)	0.047 (4)
C53	0.059 (5)	0.083 (6)	0.126 (7)	0.009 (4)	0.013 (5)	0.064 (5)
C54	0.060 (5)	0.109 (7)	0.148 (8)	0.037 (5)	0.047 (5)	0.090 (7)
C55	0.061 (4)	0.081 (5)	0.132 (7)	0.039 (4)	0.045 (5)	0.078 (5)
C56	0.072 (5)	0.106 (7)	0.156 (8)	0.062 (5)	0.075 (6)	0.089 (6)
C57	0.123 (7)	0.115 (7)	0.163 (9)	0.092 (6)	0.109 (7)	0.100 (7)
C58	0.106 (6)	0.084 (5)	0.108 (6)	0.066 (5)	0.074 (5)	0.075 (5)
C59	0.136 (7)	0.067 (5)	0.074 (5)	0.058 (5)	0.057 (5)	0.036 (4)
C60	0.127 (7)	0.080 (5)	0.069 (5)	0.058 (5)	0.043 (5)	0.037 (4)
C61	0.085 (5)	0.071 (4)	0.073 (4)	0.044 (4)	0.039 (4)	0.041 (4)
C62	0.079 (4)	0.053 (4)	0.080 (4)	0.039 (3)	0.047 (4)	0.049 (3)
C63	0.059 (4)	0.067 (4)	0.093 (5)	0.038 (3)	0.046 (4)	0.060 (4)
C64	0.044 (3)	0.041 (3)	0.055 (3)	0.019 (2)	0.015 (3)	0.020 (3)
C65	0.106 (6)	0.114 (7)	0.117 (7)	0.072 (5)	0.063 (5)	0.083 (6)
C66	0.171 (10)	0.120 (9)	0.150 (10)	0.107 (8)	0.107 (9)	0.096 (8)
C67	0.169 (12)	0.102 (9)	0.179 (13)	0.081 (9)	0.108 (11)	0.084 (9)
C68	0.077 (6)	0.057 (5)	0.141 (8)	0.016 (4)	0.060 (6)	0.005 (5)
C69	0.106 (10)	0.091 (8)	0.199 (16)	0.035 (7)	0.066 (10)	0.028 (9)
C70	0.053 (5)	0.079 (8)	0.167 (12)	0.006 (5)	0.023 (6)	−0.024 (7)
C71	0.065 (5)	0.124 (8)	0.081 (6)	0.056 (6)	−0.001 (4)	−0.012 (5)
C72	0.108 (9)	0.174 (13)	0.096 (8)	0.080 (9)	0.004 (7)	−0.027 (9)
C73	0.145 (10)	0.217 (13)	0.052 (5)	0.142 (10)	−0.005 (6)	0.023 (7)
C74	0.133 (8)	0.117 (7)	0.068 (5)	0.080 (6)	0.005 (5)	0.029 (5)
C75	0.056 (4)	0.058 (4)	0.078 (5)	0.024 (3)	0.016 (4)	−0.005 (4)
C76	0.059 (4)	0.067 (4)	0.082 (5)	0.038 (4)	0.027 (4)	0.024 (4)
C77	0.052 (4)	0.086 (5)	0.058 (4)	0.008 (3)	0.013 (3)	0.041 (4)
C78	0.139 (8)	0.072 (5)	0.064 (5)	0.019 (5)	0.025 (5)	0.027 (4)
C79	0.054 (4)	0.107 (6)	0.136 (7)	0.036 (4)	0.034 (4)	0.095 (6)
C80	0.123 (7)	0.052 (4)	0.068 (5)	0.036 (4)	0.038 (5)	0.014 (3)
Cl1	0.0892 (14)	0.1190 (17)	0.0964 (14)	0.0481 (13)	−0.0039 (11)	0.0579 (13)
Cl2	0.0820 (12)	0.0666 (11)	0.0706 (11)	0.0333 (9)	0.0200 (9)	0.0074 (9)
Cl3	0.0824 (12)	0.0665 (11)	0.1101 (15)	0.0137 (9)	0.0222 (11)	0.0511 (11)
Cl4	0.0699 (11)	0.0734 (11)	0.1299 (16)	0.0305 (9)	0.0332 (11)	0.0668 (12)
Cl5	0.0942 (14)	0.0788 (13)	0.0849 (13)	0.0273 (11)	0.0183 (11)	0.0236 (10)
Cl6	0.1023 (18)	0.227 (3)	0.168 (3)	0.079 (2)	0.0197 (17)	0.144 (3)
N1	0.050 (3)	0.051 (3)	0.054 (3)	0.028 (2)	0.014 (2)	0.022 (2)
N2	0.047 (3)	0.051 (3)	0.046 (2)	0.025 (2)	0.013 (2)	0.023 (2)
N3	0.057 (3)	0.067 (3)	0.072 (3)	0.034 (3)	0.028 (3)	0.033 (3)
N4	0.097 (5)	0.061 (4)	0.094 (4)	0.033 (3)	0.030 (4)	0.047 (3)
N5	0.065 (3)	0.059 (3)	0.053 (3)	0.024 (3)	0.014 (3)	0.015 (3)
N6	0.048 (3)	0.045 (3)	0.062 (3)	0.023 (2)	0.022 (2)	0.028 (2)
N7	0.075 (3)	0.042 (3)	0.050 (3)	0.029 (2)	0.025 (2)	0.022 (2)
N8	0.061 (3)	0.083 (4)	0.072 (4)	0.034 (3)	0.028 (3)	0.043 (3)
N9	0.048 (3)	0.043 (3)	0.051 (3)	0.018 (2)	0.013 (2)	0.020 (2)
N10	0.058 (3)	0.058 (3)	0.048 (3)	0.022 (2)	0.015 (2)	0.023 (2)
N11	0.057 (3)	0.053 (3)	0.063 (3)	0.031 (2)	0.028 (2)	0.036 (2)
N12	0.061 (3)	0.051 (3)	0.071 (3)	0.030 (2)	0.029 (3)	0.038 (3)
N13	0.071 (3)	0.056 (3)	0.059 (3)	0.036 (3)	0.032 (3)	0.034 (3)
N14	0.055 (3)	0.053 (3)	0.073 (3)	0.023 (3)	0.022 (3)	0.035 (3)
N15	0.075 (4)	0.075 (4)	0.067 (3)	0.043 (3)	0.034 (3)	0.043 (3)
N16	0.089 (4)	0.102 (5)	0.051 (3)	0.069 (4)	0.006 (3)	0.023 (3)
N17	0.055 (3)	0.075 (4)	0.083 (4)	0.032 (3)	0.020 (3)	0.042 (3)
N18	0.080 (4)	0.100 (5)	0.068 (4)	0.008 (4)	0.015 (3)	0.038 (4)
N19	0.172 (8)	0.080 (5)	0.098 (5)	0.045 (5)	0.051 (5)	0.052 (4)
N20	0.077 (4)	0.111 (6)	0.107 (5)	0.043 (4)	0.034 (4)	0.065 (5)
S1	0.0732 (11)	0.0658 (10)	0.0864 (12)	0.0430 (9)	0.0376 (9)	0.0391 (9)
S2	0.170 (3)	0.1090 (19)	0.156 (3)	0.0595 (19)	0.023 (2)	0.0920 (19)
S3	0.0772 (12)	0.0903 (13)	0.0558 (10)	0.0243 (10)	0.0240 (9)	0.0174 (9)
S4	0.0966 (14)	0.0930 (14)	0.0694 (11)	0.0398 (11)	0.0252 (10)	0.0552 (11)
S5	0.0862 (13)	0.0961 (14)	0.0681 (11)	0.0372 (11)	0.0212 (9)	0.0544 (11)
S6	0.0942 (14)	0.0870 (14)	0.0586 (11)	0.0203 (11)	0.0228 (10)	0.0282 (10)
S7	0.190 (3)	0.0782 (15)	0.0919 (17)	0.0330 (17)	0.0191 (17)	0.0413 (13)
S8	0.134 (2)	0.161 (2)	0.249 (4)	0.108 (2)	0.131 (2)	0.155 (3)
Zn1	0.0447 (4)	0.0536 (4)	0.0519 (4)	0.0203 (3)	0.0140 (3)	0.0218 (3)
Zn2	0.0542 (4)	0.0467 (4)	0.0543 (4)	0.0258 (3)	0.0218 (3)	0.0237 (3)
Zn3	0.0594 (4)	0.0540 (4)	0.0598 (4)	0.0346 (3)	0.0294 (3)	0.0351 (3)
Zn4	0.0702 (5)	0.0748 (5)	0.0585 (5)	0.0195 (4)	0.0096 (4)	0.0292 (4)

### Geometric parameters (Å, °)

**Table:**

C1—N1	1.312 (7)	C45—C46	1.426 (9)
C1—C2	1.390 (9)	C45—H45	0.9300
C1—Cl1	1.712 (7)	C46—C50	1.400 (8)
C2—C3	1.345 (10)	C46—C47	1.461 (9)
C2—H2	0.9300	C47—C48	1.337 (10)
C3—C4	1.393 (10)	C47—H47	0.9300
C3—H3	0.9300	C48—C49	1.403 (9)
C4—C5	1.422 (8)	C48—H48	0.9300
C4—C6	1.443 (10)	C49—N12	1.323 (8)
C5—N1	1.334 (7)	C49—H49	0.9300
C5—C12	1.428 (8)	C50—N12	1.362 (7)
C6—C7	1.299 (10)	C50—C51	1.441 (8)
C6—H6	0.9300	C51—N11	1.357 (7)
C7—C8	1.447 (10)	C52—N14	1.290 (8)
C7—H7	0.9300	C52—C53	1.417 (10)
C8—C9	1.402 (9)	C52—Cl5	1.730 (7)
C8—C12	1.423 (8)	C53—C54	1.377 (11)
C9—C10	1.341 (10)	C53—H53	0.9300
C9—H9	0.9300	C54—C55	1.366 (11)
C10—C11	1.397 (9)	C54—H54	0.9300
C10—H10	0.9300	C55—C63	1.390 (8)
C11—N2	1.335 (7)	C55—C56	1.427 (11)
C11—H11	0.9300	C56—C57	1.341 (12)
C12—N2	1.354 (7)	C56—H56	0.9300
C13—N3	1.147 (7)	C57—C58	1.421 (11)
C13—S1	1.611 (6)	C57—H57	0.9300
C14—N5	1.136 (7)	C58—C59	1.406 (11)
C14—S3	1.631 (7)	C58—C62	1.430 (9)
C15—N4	1.142 (9)	C59—C60	1.352 (10)
C15—S2	1.656 (8)	C59—H59	0.9300
C16—N6	1.331 (7)	C60—C61	1.391 (9)
C16—C17	1.386 (8)	C60—H60	0.9300
C16—Cl2	1.701 (6)	C61—N13	1.332 (8)
C17—C18	1.353 (9)	C61—H61	0.9300
C17—H17	0.9300	C62—N13	1.345 (7)
C18—C19	1.398 (10)	C62—C63	1.434 (9)
C18—H18	0.9300	C63—N14	1.371 (7)
C19—C20	1.384 (9)	C64—N15	1.142 (7)
C19—C26	1.421 (8)	C64—S4	1.595 (6)
C20—C21	1.363 (11)	C65—N17	1.282 (9)
C20—H20	0.9300	C65—C66	1.394 (12)
C21—C22	1.429 (10)	C65—Cl6	1.708 (9)
C21—H21	0.9300	C66—C67	1.264 (15)
C22—C23	1.359 (10)	C66—H66	0.9300
C22—C25	1.407 (8)	C67—C68	1.403 (15)
C23—C80	1.369 (11)	C67—H67	0.9300
C23—H23	0.9300	C68—C69	1.419 (15)
C24—N7	1.313 (8)	C68—C76	1.422 (11)
C24—C80	1.426 (9)	C69—C70	1.228 (18)
C24—H24	0.9300	C69—H69	0.9300
C25—N7	1.354 (8)	C70—C71	1.471 (16)
C25—C26	1.417 (9)	C70—H70	0.9300
C26—N6	1.338 (7)	C71—C72	1.363 (16)
C27—C28	1.329 (10)	C71—C75	1.419 (10)
C27—C38	1.381 (9)	C72—C73	1.347 (17)
C27—H27	0.9300	C72—H72	0.9300
C28—C29	1.435 (9)	C73—C74	1.465 (13)
C28—H28	0.9300	C73—H73	0.9300
C29—C30	1.397 (9)	C74—N16	1.375 (9)
C29—C37	1.415 (8)	C74—H74	0.9300
C30—C31	1.356 (9)	C75—C76	1.366 (10)
C30—H30	0.9300	C75—N16	1.388 (9)
C31—C32	1.417 (9)	C76—N17	1.329 (8)
C31—H31	0.9300	C77—N18	1.147 (8)
C32—C36	1.399 (7)	C77—S6	1.570 (7)
C32—C33	1.409 (9)	C78—N19	1.153 (10)
C33—C34	1.334 (9)	C78—S7	1.588 (9)
C33—H33	0.9300	C79—N20	1.142 (10)
C34—C35	1.383 (9)	C79—S8	1.628 (10)
C34—H34	0.9300	C80—H80	0.9300
C35—N9	1.336 (7)	N1—Zn1	2.404 (5)
C35—H35	0.9300	N2—Zn1	2.088 (4)
C36—N9	1.343 (7)	N3—Zn1	1.962 (5)
C36—C37	1.440 (7)	N4—Zn1	2.036 (6)
C37—N10	1.361 (7)	N5—Zn1	1.956 (5)
C38—N10	1.339 (7)	N6—Zn2	2.250 (4)
C38—Cl3	1.718 (7)	N7—Zn2	2.054 (5)
C39—N8	1.151 (7)	N8—Zn2	1.925 (5)
C39—S5	1.594 (6)	N9—Zn2	2.045 (4)
C40—N11	1.307 (7)	N10—Zn2	2.231 (5)
C40—C41	1.407 (9)	N11—Zn3	2.236 (5)
C40—Cl4	1.723 (6)	N12—Zn3	2.038 (4)
C41—C42	1.359 (9)	N13—Zn3	2.034 (5)
C41—H41	0.9300	N14—Zn3	2.216 (5)
C42—C43	1.419 (9)	N15—Zn3	1.924 (5)
C42—H42	0.9300	N16—Zn4	2.098 (6)
C43—C51	1.385 (8)	N17—Zn4	2.433 (5)
C43—C44	1.409 (9)	N18—Zn4	1.981 (6)
C44—C45	1.322 (9)	N19—Zn4	2.061 (7)
C44—H44	0.9300	N20—Zn4	1.936 (7)
			
N1—C1—C2	126.0 (6)	C54—C55—C63	116.7 (8)
N1—C1—Cl1	118.1 (5)	C54—C55—C56	122.9 (7)
C2—C1—Cl1	115.9 (6)	C63—C55—C56	120.4 (8)
C3—C2—C1	116.5 (7)	C57—C56—C55	121.0 (7)
C3—C2—H2	121.7	C57—C56—H56	119.5
C1—C2—H2	121.7	C55—C56—H56	119.5
C2—C3—C4	121.7 (7)	C56—C57—C58	121.3 (8)
C2—C3—H3	119.2	C56—C57—H57	119.4
C4—C3—H3	119.2	C58—C57—H57	119.4
C3—C4—C5	116.1 (7)	C59—C58—C57	125.1 (8)
C3—C4—C6	124.8 (7)	C59—C58—C62	116.1 (7)
C5—C4—C6	119.0 (7)	C57—C58—C62	118.9 (8)
N1—C5—C4	123.0 (6)	C60—C59—C58	122.0 (7)
N1—C5—C12	117.5 (5)	C60—C59—H59	119.0
C4—C5—C12	119.5 (6)	C58—C59—H59	119.0
C7—C6—C4	121.3 (7)	C59—C60—C61	116.9 (7)
C7—C6—H6	119.3	C59—C60—H60	121.6
C4—C6—H6	119.3	C61—C60—H60	121.6
C6—C7—C8	122.2 (7)	N13—C61—C60	125.1 (7)
C6—C7—H7	118.9	N13—C61—H61	117.4
C8—C7—H7	118.9	C60—C61—H61	117.4
C9—C8—C12	117.1 (6)	N13—C62—C58	122.4 (7)
C9—C8—C7	124.2 (6)	N13—C62—C63	118.2 (5)
C12—C8—C7	118.7 (6)	C58—C62—C63	119.4 (6)
C10—C9—C8	119.9 (6)	N14—C63—C55	123.7 (7)
C10—C9—H9	120.0	N14—C63—C62	117.2 (5)
C8—C9—H9	120.0	C55—C63—C62	119.1 (7)
C9—C10—C11	120.6 (6)	N15—C64—S4	178.7 (5)
C9—C10—H10	119.7	N17—C65—C66	123.3 (9)
C11—C10—H10	119.7	N17—C65—Cl6	118.1 (6)
N2—C11—C10	121.6 (6)	C66—C65—Cl6	118.7 (8)
N2—C11—H11	119.2	C67—C66—C65	121.6 (11)
C10—C11—H11	119.2	C67—C66—H66	119.2
N2—C12—C8	121.9 (6)	C65—C66—H66	119.2
N2—C12—C5	118.9 (5)	C66—C67—C68	118.9 (11)
C8—C12—C5	119.2 (6)	C66—C67—H67	120.5
N3—C13—S1	177.1 (6)	C68—C67—H67	120.5
N5—C14—S3	178.4 (6)	C67—C68—C69	125.0 (11)
N4—C15—S2	177.6 (7)	C67—C68—C76	116.8 (9)
N6—C16—C17	124.7 (6)	C69—C68—C76	118.2 (12)
N6—C16—Cl2	117.3 (4)	C70—C69—C68	121.8 (14)
C17—C16—Cl2	118.0 (5)	C70—C69—H69	119.1
C18—C17—C16	117.5 (7)	C68—C69—H69	119.1
C18—C17—H17	121.3	C69—C70—C71	124.2 (12)
C16—C17—H17	121.3	C69—C70—H70	117.9
C17—C18—C19	121.0 (6)	C71—C70—H70	117.9
C17—C18—H18	119.5	C72—C71—C75	121.3 (12)
C19—C18—H18	119.5	C72—C71—C70	124.1 (10)
C20—C19—C18	123.1 (7)	C75—C71—C70	114.5 (10)
C20—C19—C26	120.0 (7)	C73—C72—C71	118.9 (12)
C18—C19—C26	116.9 (6)	C73—C72—H72	120.6
C21—C20—C19	120.0 (7)	C71—C72—H72	120.6
C21—C20—H20	120.0	C72—C73—C74	123.1 (11)
C19—C20—H20	120.0	C72—C73—H73	118.4
C20—C21—C22	121.9 (6)	C74—C73—H73	118.4
C20—C21—H21	119.1	N16—C74—C73	115.6 (9)
C22—C21—H21	119.1	N16—C74—H74	122.2
C23—C22—C25	117.3 (7)	C73—C74—H74	122.2
C23—C22—C21	123.6 (7)	C76—C75—N16	119.6 (6)
C25—C22—C21	119.1 (7)	C76—C75—C71	121.6 (9)
C22—C23—C80	121.5 (7)	N16—C75—C71	118.8 (9)
C22—C23—H23	119.3	N17—C76—C75	118.8 (7)
C80—C23—H23	119.3	N17—C76—C68	121.9 (8)
N7—C24—C80	121.5 (7)	C75—C76—C68	119.3 (8)
N7—C24—H24	119.3	N18—C77—S6	179.4 (7)
C80—C24—H24	119.3	N19—C78—S7	176.2 (10)
N7—C25—C22	122.4 (6)	N20—C79—S8	178.3 (7)
N7—C25—C26	119.1 (5)	C23—C80—C24	118.0 (7)
C22—C25—C26	118.4 (6)	C23—C80—H80	121.0
N6—C26—C25	117.0 (5)	C24—C80—H80	121.0
N6—C26—C19	122.4 (6)	C1—N1—C5	116.7 (5)
C25—C26—C19	120.6 (5)	C1—N1—Zn1	133.1 (4)
C28—C27—C38	119.8 (6)	C5—N1—Zn1	110.3 (4)
C28—C27—H27	120.1	C11—N2—C12	118.9 (5)
C38—C27—H27	120.1	C11—N2—Zn1	121.6 (4)
C27—C28—C29	120.8 (6)	C12—N2—Zn1	119.4 (4)
C27—C28—H28	119.6	C13—N3—Zn1	174.7 (5)
C29—C28—H28	119.6	C15—N4—Zn1	162.7 (7)
C30—C29—C37	119.8 (6)	C14—N5—Zn1	172.0 (5)
C30—C29—C28	125.2 (6)	C16—N6—C26	117.5 (5)
C37—C29—C28	115.0 (6)	C16—N6—Zn2	131.5 (4)
C31—C30—C29	121.5 (6)	C26—N6—Zn2	111.0 (4)
C31—C30—H30	119.3	C24—N7—C25	119.3 (6)
C29—C30—H30	119.3	C24—N7—Zn2	124.8 (5)
C30—C31—C32	120.6 (6)	C25—N7—Zn2	115.8 (4)
C30—C31—H31	119.7	C39—N8—Zn2	166.5 (5)
C32—C31—H31	119.7	C35—N9—C36	118.1 (5)
C36—C32—C33	117.2 (5)	C35—N9—Zn2	125.2 (4)
C36—C32—C31	119.8 (6)	C36—N9—Zn2	116.7 (4)
C33—C32—C31	123.0 (6)	C38—N10—C37	116.7 (5)
C34—C33—C32	118.8 (6)	C38—N10—Zn2	132.9 (4)
C34—C33—H33	120.6	C37—N10—Zn2	110.0 (4)
C32—C33—H33	120.6	C40—N11—C51	117.2 (5)
C33—C34—C35	121.2 (6)	C40—N11—Zn3	131.8 (4)
C33—C34—H34	119.4	C51—N11—Zn3	110.9 (4)
C35—C34—H34	119.4	C49—N12—C50	118.3 (5)
N9—C35—C34	121.7 (6)	C49—N12—Zn3	125.4 (4)
N9—C35—H35	119.2	C50—N12—Zn3	116.3 (4)
C34—C35—H35	119.2	C61—N13—C62	117.5 (5)
N9—C36—C32	122.8 (5)	C61—N13—Zn3	126.2 (4)
N9—C36—C37	117.7 (5)	C62—N13—Zn3	116.2 (4)
C32—C36—C37	119.4 (5)	C52—N14—C63	117.4 (6)
N10—C37—C29	124.0 (5)	C52—N14—Zn3	132.9 (5)
N10—C37—C36	117.3 (5)	C63—N14—Zn3	109.7 (4)
C29—C37—C36	118.7 (6)	C64—N15—Zn3	167.6 (5)
N10—C38—C27	123.7 (6)	C74—N16—C75	122.3 (7)
N10—C38—Cl3	116.4 (5)	C74—N16—Zn4	118.9 (6)
C27—C38—Cl3	119.9 (5)	C75—N16—Zn4	118.7 (5)
N8—C39—S5	178.3 (6)	C65—N17—C76	117.5 (7)
N11—C40—C41	123.7 (6)	C65—N17—Zn4	132.5 (5)
N11—C40—Cl4	118.3 (5)	C76—N17—Zn4	109.9 (5)
C41—C40—Cl4	118.1 (5)	C77—N18—Zn4	175.0 (7)
C42—C41—C40	118.4 (6)	C78—N19—Zn4	171.6 (8)
C42—C41—H41	120.8	C79—N20—Zn4	170.4 (7)
C40—C41—H41	120.8	N5—Zn1—N3	120.0 (2)
C41—C42—C43	120.2 (6)	N5—Zn1—N4	93.1 (2)
C41—C42—H42	119.9	N3—Zn1—N4	101.1 (2)
C43—C42—H42	119.9	N5—Zn1—N2	130.19 (19)
C51—C43—C44	120.5 (6)	N3—Zn1—N2	106.42 (19)
C51—C43—C42	115.9 (6)	N4—Zn1—N2	94.7 (2)
C44—C43—C42	123.5 (6)	N5—Zn1—N1	87.35 (19)
C45—C44—C43	121.5 (6)	N3—Zn1—N1	91.93 (18)
C45—C44—H44	119.2	N4—Zn1—N1	164.6 (2)
C43—C44—H44	119.2	N2—Zn1—N1	73.48 (16)
C44—C45—C46	120.3 (6)	N8—Zn2—N9	125.3 (2)
C44—C45—H45	119.8	N8—Zn2—N7	123.9 (2)
C46—C45—H45	119.8	N9—Zn2—N7	110.69 (18)
C50—C46—C45	120.2 (6)	N8—Zn2—N10	93.2 (2)
C50—C46—C47	116.1 (6)	N9—Zn2—N10	77.90 (18)
C45—C46—C47	123.8 (6)	N7—Zn2—N10	101.06 (19)
C48—C47—C46	119.4 (6)	N8—Zn2—N6	93.5 (2)
C48—C47—H47	120.3	N9—Zn2—N6	96.30 (16)
C46—C47—H47	120.3	N7—Zn2—N6	77.01 (18)
C47—C48—C49	120.0 (7)	N10—Zn2—N6	172.90 (16)
C47—C48—H48	120.0	N15—Zn3—N13	126.8 (2)
C49—C48—H48	120.0	N15—Zn3—N12	123.9 (2)
N12—C49—C48	123.1 (7)	N13—Zn3—N12	109.31 (19)
N12—C49—H49	118.5	N15—Zn3—N14	94.9 (2)
C48—C49—H49	118.5	N13—Zn3—N14	78.6 (2)
N12—C50—C46	123.2 (6)	N12—Zn3—N14	97.11 (18)
N12—C50—C51	118.3 (5)	N15—Zn3—N11	93.3 (2)
C46—C50—C51	118.5 (6)	N13—Zn3—N11	96.81 (19)
N11—C51—C43	124.6 (6)	N12—Zn3—N11	77.92 (18)
N11—C51—C50	116.5 (5)	N14—Zn3—N11	171.87 (17)
C43—C51—C50	118.9 (6)	N20—Zn4—N18	121.6 (3)
N14—C52—C53	123.8 (7)	N20—Zn4—N19	99.9 (3)
N14—C52—Cl5	116.9 (5)	N18—Zn4—N19	93.8 (3)
C53—C52—Cl5	119.3 (7)	N20—Zn4—N16	110.0 (3)
C54—C53—C52	117.0 (8)	N18—Zn4—N16	124.5 (2)
C54—C53—H53	121.5	N19—Zn4—N16	96.3 (3)
C52—C53—H53	121.5	N20—Zn4—N17	90.2 (2)
C55—C54—C53	121.4 (8)	N18—Zn4—N17	87.7 (2)
C55—C54—H54	119.3	N19—Zn4—N17	167.1 (3)
C53—C54—H54	119.3	N16—Zn4—N17	72.5 (2)
